# Gait Study of Parkinson’s Disease Subjects Using Haptic Cues with A Motorized Walker

**DOI:** 10.3390/s18103549

**Published:** 2018-10-19

**Authors:** Minhua Zhang, N. Sertac Artan, Huanying Gu, Ziqian Dong, Lyudmila Burina Ganatra, Suzanna Shermon, Ely Rabin

**Affiliations:** 1 College of Engineering and Computing Sciences, New York Institute of Technology, New York, NY 10023, USA; mzhang16@nyit.edu (M.Z.); nartan@nyit.edu (N.S.A.); hgu03@nyit.edu (H.G.); 2 College of Osteopathic Medicine, New York Institute of Technology, 101 Northern Blvd, Glen Head, NY 11545, USA; lburina@nyit.edu (L.B.G.); sshermon@nyit.edu (S.S.); ely_rabin@sciarc.edu (E.R.)

**Keywords:** Parkinson’s Diseases, motorized walker, haptic cue, gait pattern, statistics study

## Abstract

Gait abnormalities are one of the distinguishing symptoms of patients with Parkinson’s disease (PD) that contribute to fall risk. Our study compares the gait parameters of people with PD when they walk through a predefined course under different haptic speed cue conditions (1) without assistance, (2) pushing a conventional rolling walker, and (3) holding onto a self-navigating motorized walker under different speed cues. Six people with PD were recruited at the New York Institute of Technology College of Osteopathic Medicine to participate in this study. Spatial posture and gait data of the test subjects were collected via a VICON motion capture system. We developed a framework to process and extract gait features and applied statistical analysis on these features to examine the significance of the findings. The results showed that the motorized walker providing a robust haptic cue significantly improved gait symmetry of PD subjects. Specifically, the asymmetry index of the gait cycle time was reduced from 6.7% when walking without assistance to 0.56% and below when using a walker. Furthermore, the double support time of a gait cycle was reduced by 4.88% compared to walking without assistance.

## 1. Introduction

Individuals with Parkinson’s disease (PD) may suffer from movement disorders [1]. The initial symptoms include involuntary tremors of hands, arms, or legs, slow movement, rigidity, and postural instability. These symptoms lead to different gait disturbances [2]. Stolze et al. found that people with PD had a significant spatiotemporal parameters reduction in step length and walking velocity compared with healthy individuals [3]. Individuals with PD may also experience difficulties in step initiation and in postural changes [4]. Although dopaminergic medications, which increase the levels of dopamine in the brain, may help improve gait, their effectiveness decreases as the disease progresses [5].

A growing body of research has demonstrated that individuals with PD can benefit from various cueing devices [6,7,8]. Individuals with PD increased their pedaling rate under auditory cueing (provided by a metronome) and visual cueing (presented as central road markers) conditions [9,10]. Individuals with PD can benefit from haptic (touch and proprioception) feedback to improve balance. Haptic cues from the use of a walking stick [11], and visual cues from a laser cane reduce forward/backward and side to side movements [6]. Gait patterns of PD patients walking straight on a level ground without assistance were well investigated [2,4,12,13].

The aim of this study is to investigate the immediate gait modifications of individuals with PD when they switch from walking without assistance to walking with a conventional and a motorized walker. We attempt to answer two questions: (1) Can haptic cues help improve the motor performance of patients? and (2) how effectively do PD subjects adapt to various speed cues?

Our analysis showed that test subjects walking with a conventional walker and a motorized walker showed better gait symmetry performance than walking without assistance. Subjects also walked faster with an increasing haptic speed cue and increased stride height and stride length while using the motorized walker with a speed cue above the medium speed range. We also observed that test subjects walking with a conventional walker and a motorized walker exhibited less double support time out of the gait cycle time. When walking with the motorized walker on a medium speed cue, subjects had on average 4.88% less double support time, which indicated a faster gait initiation under this condition.

## 2. Related Work

Human gait is the periodic movement of limbs, trunk, and arms during locomotion. The bipedal gait cycle consists of right-side and left-side steps. De Rossi et al. introduced a six-phase gait model, where each side has an initial, a swing, and a stance phase [14]. An eight-phase gait model was introduced that expands the initial phase into two additional sub phases: Initial contact and loading response phases [15]. Gait cycle time, stride length, stride height, gait initiation, and other gait parameters are of interest to clinicians in understanding the disease progression of patients with PD [13,16]. The stance phase for the control subjects occupies approximately 60% of the gait cycle, and the swing phase occupies the remaining 40% [17]. Individuals with PD have difficulty controlling balance and gait, which can lead to falling, injury, dependence, and loss of quality of life. Individuals with PD have shorter steps, reduced stride height, and extended stance phase compared to the healthy controls [18]. Impaired balance and gait, including freezing of gait, in PD has been attributed in part to changes in attention. Freezing of gait often occurs during situations requiring gait changes or divided attention such as turning or narrow passages [19]. PD subjects who experience freezing of gait have distinctive impairments in the bilateral coordination of locomotion [20].

External spatial and temporal cues help people with PD to overcome slowing of their gate. Auditory timing cues can have positive rehabilitative effects on various gait characteristics of PD [21], stroke [22], and hemiparesis [23] patients. For patients with PD, visual cues have shown to improve stride length, while auditory cues have shown to improve cadence [21]. However, the beneficial effects on gait can disappear when the visual and attentional cues are removed [24]. Thus, the cues should always be present to maintain their rehabilitative effects. Haptic cues from non-supportive manual contact with an external surface provides somatosensory information that individuals with poor balance improve the control of upright standing during intervention programs [25,26]. Such cues from assistive ambulatory devices such as walking canes and walkers might also improve stability and orientation during gait, but at the cost of reducing walking speed. People with PD walked with slower gait speed and reduced stride length when using a cane and a wheeled walker compared to walking without any device [27]. However, PD subjects produced natural gait pattern when using a wheeled walker, by not slowing velocity or increasing variability as other devices do [28]. In our present study, we tested whether gait of people with PD would improve when following haptic speed cues from a self-propelled walker, self-navigating walker.

## 3. Methods

In this study, we collected spatiotemporal postural and gait data from six PD subjects walking in a predesigned course via the VICON motion capture system (Vicon, Denver, CO, USA) under three conditions of manual gait aids: none (without assistance), with a conventional rolling walker, and with a motorized walker, where the motorized walker can be configured to operate at three different speed ranges: Low (32–52 cm/s), medium (52–72 cm/s), and high (72–96 cm/s). The speed is measured without any payload, where the walker’s movement is propelled by its motors without users holding on to it to move around. We applied statistical analysis on the extracted gait features to determine the significance of the gait modifications and used asymmetry index [29] to analyze the bilateral coordination of the locomotion.

### 3.1. Subjects and Protocols

Six PD patients (five males and one female) between the ages of 44 and 77 (median: 66) and at Hoehn and Yahr stages 2–3, were recruited at the New York Institute of Technology College of Osteopathic Medicine to participate in this study. This study was reviewed and approved by New York Institute of Technology (NYIT) Institutional Review Board.The Unified Parkinson’s Disease Rating Scale (UPDRS) scores for the subjects ranged from 18 to 33, and Mini-Mental State Examination (MMSE) scores ranged from 26 to 30. Years diagnosed was between 1 and 27 years (median: 24). Each patient was instructed to walk in a preset course for 5 meters including a 90-degree turn under the following haptic cue conditions: (1) Without assistance, (2) with a conventional walker, and (3) with a motorized walker with various speed cues.

**Task:** With each of the three experimental conditions, patients walked alongside a 25-foot board, then proceeded to make a 90 degree turn, and continued walking alongside another 25-foot board. The two boards were at a right angle to each other, and the patients walked on the left side of each board.

For each patient, two to three trials of walking without assistance, two to three trials of walking with a conventional walker, and six to ten trials of walking with a motorized walker were recorded. Incomplete trials were excluded from the study. Notations representing each trial are listed in Table 1. The speed configurations of the walker in this table are measured at zero load, which indicates that the walker is moving untethered, i.e., without having a patient holding on to it.

### 3.2. Apparatus

**Figure 1:**
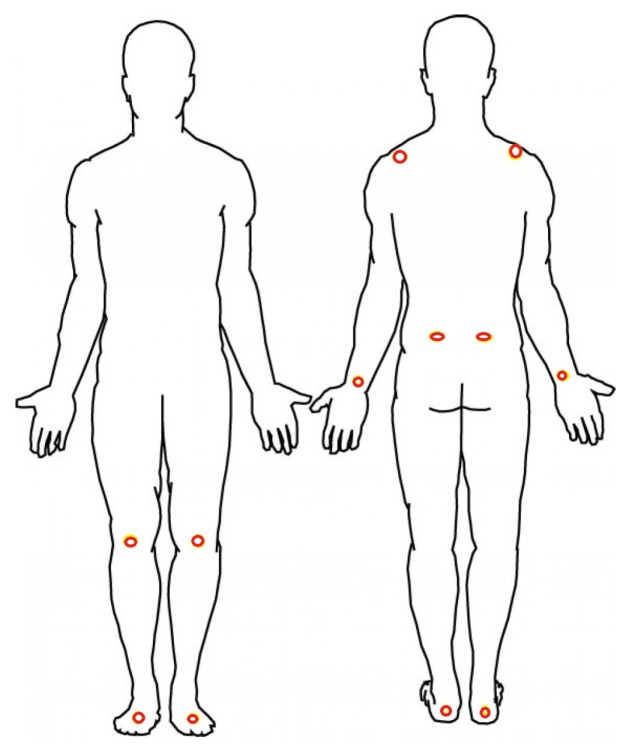
Red circles show the location of the retroreflective markers.

A nine-camera VICON motion capture system (Vicon, Denver CO) with a sampling rate of 100 Hz was used for recording the gait and postural parameters of the subjects by measuring ongoing position of reflective markers attached to the following body landmarks: Bilateral metatarsals, achilles tendons, lateral collateral ligaments of the knees, iliac crests, wrists, and acromions as shown in Figure 1. Two additional markers were placed on each walker.

**Figure 2:**
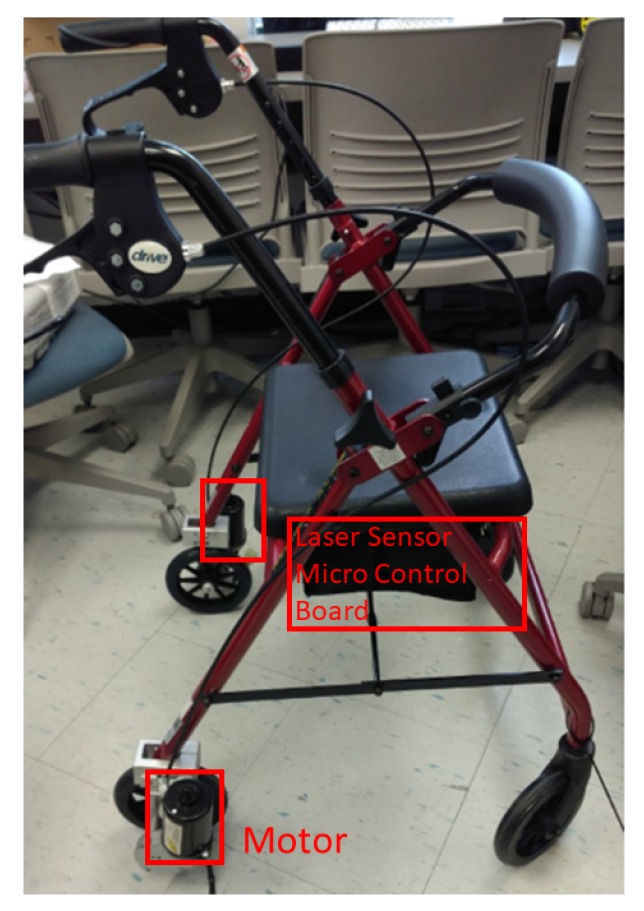
The motorized walker with speed control, preset course navigation, and obstacle avoidance.

A motorized walker as shown in Figure 2 was designed to provide speed control and navigation in a preset course [30] by instrumenting a conventional walker with two 64 mm, 12 V gear head motors (Am Equipment, Jefferson, OR) on the rear wheels, a URG-04LX-UG01 laser range sensor (Hokuyo Osaka, Japan, 2015), and a micro-controller board (stored inside the compartment under the walker seat).

The motorized walker was configured to move forward and turn at various pre-set speeds. When using the walker, the user holds the handles of the walker where a haptic cue is provided with the automatic movement of the walker that leads the user to move and turn at a pre-set speed. Table 2 shows the various speed configurations for the motorized walker. As an example, let us consider the m01 trial, i.e., the first trial with the motorized walker. The motorized walker accelerates up to the maximum speed of 32 cm/s. The average acceleration is 24 cm/s^2^ and the acceleration time is 0.06 s to reach the maximum speed. The configuration parameters can be changed depending on the movement ability of PD patients. In this study, some patients had trials with up to the 80 cm/s maximum haptic speed cue. Upon sensing an obstacle in its path, as a safety measure, the motorized walker proportionally decreases its speed and comes to a full stop.

### 3.3. Data Analysis

**Figure 3:**
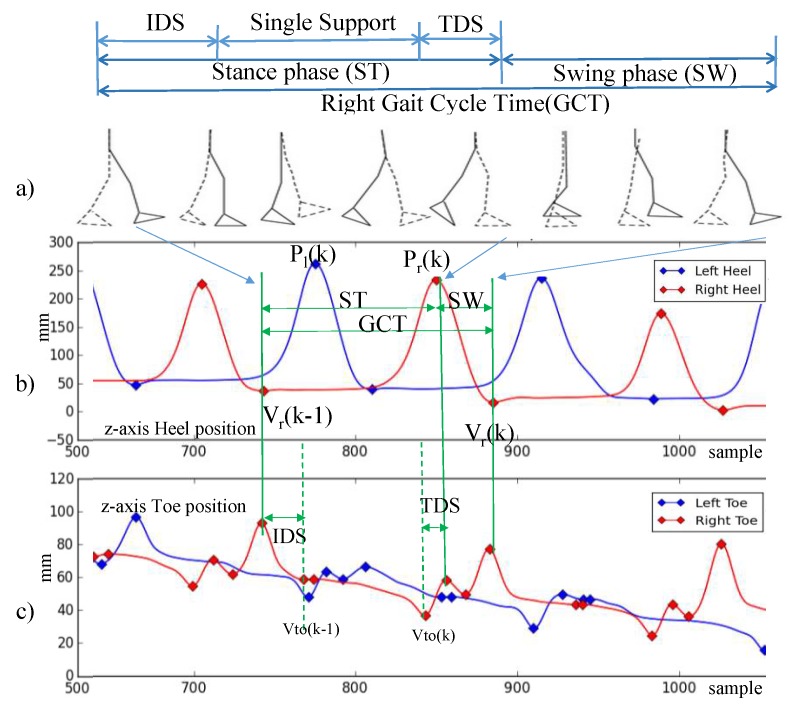
Gait cycle model. (**a**) Gait cycle model (**b**) z-axis heel position (**c**) z-axis toe position.

Gait is a complex sensorimotor activity that involves spatiotemporal coordination of the legs, trunk, arms, and dynamic equilibrium, all of which are affected by PD. Table 3 outlines the terminology used in the description of the gait model. The duration of a complete gait cycle is defined as the *gait cycle time* (GCT) [14,31] and shown in Figure 3a. GCT is divided into two phases: *Stance time* (ST) and *swing time* (SW). ST denotes the duration when the foot is on the floor, while SW denotes the duration when the foot is in the air. *Double support* (DS) denotes the period when both feet are on the floor. DS can be divided into *initial double support* (IDS), which denotes the duration between the initial foot’s heel contact and the other foot’s toe off, and *terminal double support* (TDS), which denotes the duration of the subsequent opposite-side heel contact and toe off [32].

The gait parameters are calculated based on the spatiotemporal measurement of the marker locations attached on the subject’s body. As shown in Figure 3b,c, we use the vertical heel and toe positions to identify the gait phases. We use the following spatial location measurements in identifying gait events and calculating gait parameters: V(k) denotes the $k^{th}$ valley of the heel position in z-axis, P(k) denotes the $k^{th}$ peak of the heel position in z-axis, and $V_{to}$ denotes the nearest valley of the toe position in z-axis.

Gait Cycle Time (GCT) is calculated as the duration between two consecutive valleys of the heel position as:

(1) GCT(k)=V(k)−V(k−1)

Swing Time (SW) is calculated as the duration between two consecutive valley and peak of the heel position:

(2) SW(k)=V(k)−P(k)

Stance Time (ST) is the remaining period of a GCT minus swing time:

(3) ST(k)=GCT(k)−SW(k)

Initial Double Support (IDS) time is the duration between the valley of the heel position and its nearest valley of the toe position:

(4) $$\mathrm{IDS}\left(k\right)=V_{to}-V\left(k\right)$$

Terminal Double Support (TDS) time is the duration between the peak of the heel position and its nearest valley of the toe position:

(5) $$\mathrm{TDS}\left(k\right)=P\left(k\right)-V_{to}$$

Step height is the difference between the peak of the heel position and its nearest valley position:

(6) SH(k)=P(k)−V(k−1)

Step length is defined as the difference between the x coordinate of the heel position between two consecutive peaks:

(7) $$\mathrm{SL}\left(k\right)=P_{x}\left(k\right)-P_{x}\left(k-1\right)$$

where the subscript *x* indicates the *x* coordinate. Finally, velocity is defined as the ratio of step length over gait cycle time:

(8) $$\mathrm{Vel}=\frac{\mathrm{SL}}{\mathrm{GCT}}$$

Previous studies showed that the ratio of stance/swing of healthy subjects is about 3:2 [33,34]. IDS warrants the upright stability during walking [35]. It reduces to zero when a subject is running, which means both feet are airborne twice during the gait cycle [36]. Sofuwa et al. [37] also showed that PD patients have decreased gait speed and stride length and increased double support time.

Morris et al. [38] reported that patients in the earlier stages of PD may have extended stance time which allows PD subjects maintain their gait stability. The IDS may increase in the the late stage of PD. This long IDS can give the impression that the PD subjects *glue* their feet on the ground.

## 4. Signal Processing for Gait Analysis

**Figure 4:**
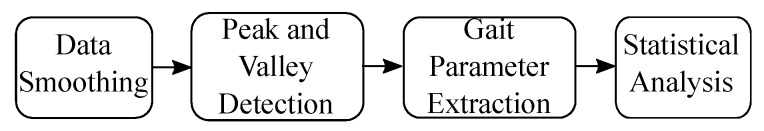
Signal processing procedure for gait analysis.

In this Section, we introduce the signal processing procedure for gait signal analysis. A block diagram of the procedure is outlined in Figure 4.

First, we smoothed raw data to remove noise and identify gait cycle based on [39] through peak and valley detections. Then, we extracted the gait parameters following the definition in Section 3. Finally we studied the statistical significance of the observations. We explain each procedure in detail in the following section.

### 4.1. Data Smoothing

To remove noise in the measured signal to find peaks and valleys, two types of filters were evaluated for data smoothing: (1) Convolution [40], and (2) Savitzky-Golay low-pass filter [41]. Convolution did not decrease the amplitude of the signal and retained more of the gait details, and in general performed better than Savitzky-Golay low-pass filter in this context. Thus, we chose convolution for smoothing. A 40-sample Hanning window is used for convolution, so that the window size is less than half of the gait cycle time (0.5 s).

### 4.2. Peak and Valley Detection and Principle Gait Parameters Extraction

**Figure 5:**
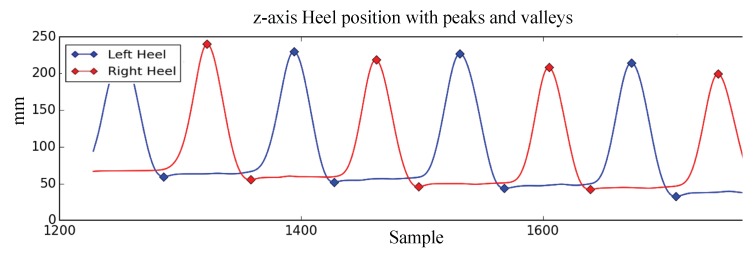
Peaks and valleys of z-axis heel position.

We implemented the peak and valley detection algorithm in Python based on the algorithm presented by Ferrari et al. [42]. We used the *argrelextrema* function from the SciPy Python library’s signal processing toolbox [43] to identify the peak and valley candidates. Portions of the data that correspond to the turning phase might still be mistaken as peaks and valleys. To remove the turning phase peaks and valleys detection errors, only one peak between two valleys and only one valley between two peaks were selected. Figure 5 shows the peaks and valleys identified after the smoothing operation is completed and turning phase peaks and valleys are removed. Once the peaks and valleys are identified SW, ST, IDS, SL, and SH are calculated using (2)–(7).

### 4.3. Statistical Analysis

In this study, we are interested in the variability among different sets of trials when subjects walk without assistance, with a conventional walker, and with a motorized walker providing haptic speed cues. Towards that goal, we evaluated the mean and standard deviation from the five sets of trials (c, ml, mm, mh, w) and applied statistical hypothesis testing. First, we apply Shapiro-Wilk test to verify that the data follow normal distribution (*p* > 0.05), then we applied t-test (alpha = 0.05) to test the null hypothesis that the gait parameters do not vary whether the patient is walking without assistance, or using a conventional walker or a motorized walker.

## 5. Results

In this Section, we present the results comparing the gait parameters observed at different trials and analyze gait symmetry and individual gait performance.

### 5.1. Gait Parameters

Table 4 shows the spatiotemporal gait parameters for all subjects measured (mean ± SD) for different trials, i.e., walking without assistance (c), walking with a conventional walker (w), and walking with the motorized walker (m) with low (ml), medium (mm), and high (mh) speed cues. The subjects’ walking speed follows the cueing speed of the motorized walker. More specifically, PD subjects walking velocity is 29.24±7.94 cm/s on low speed cues; 52.80±10.56 cm/s on medium speed cues; and 67.33±11.67 cm/s on high speed cues.

We observed that the stride height and stride length also increase as the cueing speed increases. At the lowest cueing speed, the subjects have the lowest stride height (SH: 14.52±4.09 cm) and shortest stride length (SL: 49.72±12.58 cm). At the highest cueing speed, the subjects have the highest stride height (SH: 21.08±2.97 cm) and length (SL: 74.76±12.11 cm).

In Table 5, we show that there was a significant difference in walking patterns for subjects walking with the motorized walker with medium speed cue (mm) and walking without any assistance (c) in all gait parameters (*p* < 0.05) with the exception of IDS (*p* = 0.059). Conversely, the difference is insignificant between walking without assistance and motorized walker with high speed cue (mh), and conventional walker (w) in all gait parameters with the following exceptions for both cases: SW, SL, and Vel, where the differences are significant (*p* < 0.05). These results suggest that motorized walker at medium speed cues has the largest gait modification in PD subjects.

Table 6 summarizes the ratio of ST, IDS, and TDS periods in a gait cycle. Accordingly, the motorized walker reduces the PD subjects’ ST over GCT when the speed cues increase (74.40%, 73.10%, 71.53% respectively in ml, mm, mh). This suggests that PD subjects use less time on the ground when the speed cue increases. PD subjects walking without assistance present higher IDS and TDS to GCT ratios (IDS/GCT=18.75%, TDS/GCT=16.88%), while PD subjects walking with motorized walker on medium speed cues (mm) have lower ratios (IDS/GCT=15.66%, TDS/GCT=15.09%). This corresponds to 3.09% and 1.79% lower IDS/GCT, and TDS/GCT ratios, respectively. These observations may indicate PD subjects have less hesitation in initiating a step when walking with motorized walker on medium speed cues.

### 5.2. Gait Symmetry

Gait symmetry is defined as the perfect agreement between the actions of the lower limbs [44]. *Asymmetry index*, denoted as $I_{a}$ can be used to quantify gait symmetry or asymmetry [29]:

(9) $$I_{a}=\frac{X_{L}-X_{R}}{\max(X_{L},X_{R})}\times 100$$

where, X∈[GCT,SH,SL,Vel], and subscripts *L* and *R* represent left side and right side, respectively. $I_{a}\in \left[-1,0\right)$ represents right asymmetry (i.e., the value of the gait parameter is higher on the right side), and $I_{a}\in \left(0,1\right]$ represents left asymmetry. $I_{a}=0$ when there is no asymmetry.

Table 7 shows the asymmetry indices of gait parameters. Our results indicate that PD subjects exhibit better overall gait symmetry when they use a motorized or conventional walker compared to walking without assistance. The GCT asymmetry indices ($I_{a,GCT}$) of motorized walker (below 0.1 to 0.56%) or conventional walker (0.53%) are much lower than walking without assistance (6.7%).

For the stride height asymmetry index ($I_{a,SH}$), similarly, the subjects have more symmetric foot-raising posture with either of the walkers compared to walking without assistance (5.7%). Subjects using the conventional walker (1.48%) show better stride height symmetry compared to using the motorized walker (between −3.99 and 2.12%). For the stride length and velocity asymmetry index ($I_{a,SL}$, $I_{a,\mathrm{Vel}}$), subjects using the motorized walker with medium and high speed cues show better symmetry with regards to stride length ($I_{a,SL}$) at 1.41% and 1.33%, respectively compared to walking without assistance and walking with the conventional walker.

### 5.3. Individual Gait Performance

In this Section, we study the individual PD subject’s (P1–P6) trials and compare the results of gait performance for each individual. To determine whether cue speed affects (1) the quality of gait performance matching the cue speed and/or (2) amelioration of PD gait symptoms, we organize the trials such that, for each subject, the speed cue starts at a low speed, and gradually increases to higher speeds.

**Figure 6:**
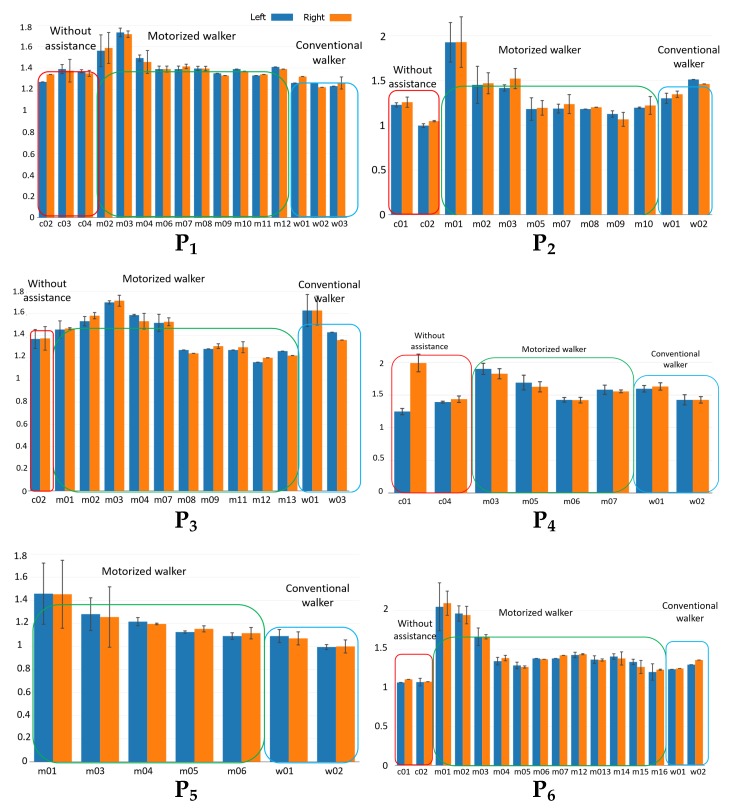
Gait cycle time (GCT) for each of the 6 subjects ($P_{1}$–$P_{6}$) without assistance, with motorized walker, and with conventional walker.

Figure 6a–f show the GCT for each subject for different trials (based on the notation introduced in Table 1). Each bar corresponds to the mean GCT value for a different trial in seconds. The blue bars represent the mean values for the left side, orange bars represent the mean values for the right side, and the error bars indicate the variance of GCT for each case. Trials with noisy or corrupted data due to the data acquisition issues are excluded. The individual GCT bar chart indicates PD subjects need time to adapt to the motorized walker. We observe that PD subjects have high GCT and GCT variance when they start to use the motorized walker. However, after the first one to three trials, GCT drops to a relatively lower level and fluctuates in a smaller range. For instance, for subject P1, GCT ∈[1.5,1.7] during the first three trials using the motorized walker, but drops to [1.3,1.4] after that.

## 6. Discussion

In this study, we observed that the study subjects using a motorized walker exhibit a walking pattern with more symmetric performance on both sides. The study subjects’ walking velocity increased as the speed cue increased. We also observed that study subjects required a few trials to get used to the motorized walker. While initial trial shows large variabilities, as the number of trials increases, GCT variability decreases. Our study provides further evidence that PD subjects exhibit a natural gait pattern, when using a wheeled walker without slowing down [28]. The subjects’ gait performance under different haptic speed cues also provide insights on immediate gait modification under these speed cues conditions.

Although, with six subjects, the current study can provide some insights, a wider perspective can be achieved if the sample size is increased in a future study. In particular, more subjects at each PD stage, and more subjects based on years from diagnosis and age can help evaluate the efficacy of the motorized walker at different stages of the disease.

The availability of a large group of subjects can also enable protocols that can evaluate parameters at higher granularity. Another possible extension to this work is to reverse the order of the speed cues, starting from higher speed cues to lower speeds to see the impact on the adaptation of using the motorized walker. Conversely, speed cues can also be randomized to investigate the impact of initial cues to the overall adaptation.

Hausdorff et al. [20,45] have proposed that gait control impairments (gait asymmetry, and bilateral dyscoordination), even during periods in which freezing is not present, set the stage for the occurrence of a freezing of gait (FOG) episode. Our study shows that the walker can immediately modify the gait regulation of PD subjects, demonstrating more bilateral gait symmetry. In this case, it can be hypothesized that the motorized walker providing haptic cues can possibly improve the bilateral coordination of locomotion and can potentially reduce the FOG occurrence in PD subjects. Future work with a long-term use of the motorized walker will allow enough FOG episodes to be observed. In parallel, new analytic models should be developed to provide quantitative analysis of gait performance to evaluate the efficacy of the intervention.

## 7. Conclusions

We studied the immediate gait modifications of individuals with Parkinson’s disease, comparing between walking without assistance to walking with a conventional and a self-navigating motorized walker that provides haptic speed cues. We observed that the subjects’ gait exhibited more symmetry with reduced initial double support time when walking with a walker compared to walking without assistance. When using a motorized walker with medium speed cues, subjects’ IDS and TDS ratios to GCT were reduced by 3.09%, and 1.78% compared to walking without assistance, respectively, with an overall 4.88% reduction in double support time. During the individual analysis, we observed a learning curve for the subjects to get used to the motorized walker. This usually took one to three trials, after which GCT variability was reduced. The test subjects’ walking velocity was strongly related to the speed cues. When the cueing speed increased, we observed a decrease in the GCT, SW, ST, and IDS, while SL, SH, and velocity increased. Reduced step initiation time and increased stride length and height indicated improvement in gait for the test subjects. The gait improvements indicate the test subjects exhibit a gait pattern closer to healthy controls with better gait symmetry and balance. More symmetry in bilateral locomotion is promising for improved balance of the subjects in reducing potential fall risks. The motorized walker can be adopted as a rehabilitation device in gait training. The use of a customized motorized walker also has potential to mitigate the freezing of gait episodes by providing continuous haptic cues for subjects to follow without divided attention.

## Figures and Tables

**Table 1:** Trial notations.

Notation	Definition
c	walking without assistance
mxx	walking with motorized walker, trial number XX
ml	walking with motorized walker, low speed cue: [32,52) cm/s
mm	walking with motorized walker, medium speed cue: [52,72) cm/s
mh	walking with motorized walker, high speed cue: [72,96) cm/s
w	walking with conventional walker

**Table 2:** Speed settings for trials with the motorized walker.

Trial	Speed	Max Speed	Acceleration	Accel. Time
No.	Range	[cm/s]	[cm/s${}^{2}$]	[s]
m01	ml	32	24	0.06
m02	ml	44	24	0.06
m03	mm	52	24	0.06
m04	mm	60	20	0.1
m05	mm	64	20	0.1
m06	mh	72	12	0.2
m07	mh	80	12	0.2

**Table 3:** Terminology.

Terminology	Definition
GCT	Gait Cycle Time
SW	Swing Time
ST	Stance Time
DS	Double Support
IDS	Initial Double Support
TDS	Terminal Double Support
SH	Step Height
SL	Step Length

**Table 4:** Mean and standard deviation of gait parameters for Parkinson’s disease (PD) subjects walking without assistance (c), with conventional walker (w), and with motorized walker (m) at low (ml), medium (mm), and high (mh) speed cues.

Gait Parameters			m		
(unit)	c	w	ml	mm	mh
GCT (s)	1.29±0.25	1.34±0.21	1.68±0.28	1.4±0.2	1.31±0.13
SW (s)	0.35±0.05	0.39±0.06	0.43±0.10	0.38±0.06	0.38±0.06
ST (s)	0.94±0.22	0.95±0.2	1.25±0.28	1.02±0.17	0.94±0.1
IDS (s)	0.24±0.69	0.24±0.11	0.28±0.14	0.22±0.01	0.21±0.06
TDS (s)	0.22±0.03	0.22±0.12	0.25±0.13	0.21±0.13	0.21±0.05
SL (cm)	92.98±1.24	70.61±27.70	49.72±12.58	68.59±11.86	74.76±12.11
SH (cm)	21.19±3.14	20.78±3.40	14.52±4.09	19.02±3.31	21.08±2.97
Vel (cm/s)	75.28±12.99	65.89±17.36	29.24±7.94	52.80±10.56	67.33±11.67

**Table 5:** *p*-values for the pairwise comparison of gait parameters for PD subjects walking without and with motorized walker.

Gait Parameters	Pairwise Comparison of *p*-Value		
(unit)	c vs. mm	c vs. mh	c vs. w
GCT (s)	0.006	0.459	0.245
SW (s)	0.014	0.025	0.002
ST (s)	0.016	0.959	0.686
IDS (s)	0.059	0.754	0.216
TDS (s)	0.004	0.142	0.154
SL (cm)	≪0.01	0.001	≪0.01
SH (cm)	0.001	0.866	0.583
Vel (cm/s)	≪0.01	0.002	0.07

**Table 6:** The ratio of stance time (ST), initial double support (IDS), and terminal double support (TDS) in a gait cycle.

Gait Parameters		m			
	c	ml	mm	mh	w
ST/GCT	72.76%	74.40%	73.10%	71.53%	70.98%
IDS/GCT	18.75%	16.78%	15.66%	15.72%	17.48%
TDS/GCT	16.88%	14.94%	15.09%	16.18%	16.59%
IDS/ST	25.77%	22.56%	21.43%	21.98%	24.63%
TDS/ST	22.45%	20.08%	20.65%	22.62%	23.37%

**Table 7:** Asymmetry indices for straight walking.

Gait Parameters		m			
	c	ml	mm	mh	w
$I_{a,GCT}$	6.7%	0.56%	<0.1%	<0.1%	0.53%
$I_{a,SH}$	5.7%	−3.99%	3.46%	2.12%	1.48%
$I_{a,SL}$	−1.8%	−2.10%	1.44%	1.33%	−3.25%
$I_{a,\mathrm{Vel}}$	3.4%	−9.50%	1.48%	2.03%	−2.75%
